# Association analyses of the MAS-QTL data set using grammar, principal components and Bayesian network methodologies

**DOI:** 10.1186/1753-6561-5-S3-S8

**Published:** 2011-05-27

**Authors:** Burak Karacaören, Tomi Silander, José M Álvarez-Castro, Chris S Haley, Dirk Jan de Koning

**Affiliations:** 1 The Roslin Institute and R(D)SVS, University of Edinburgh, EH25 9PS, Roslin, UK; 2 Tomi Silander,A*STAR Institute of High Performance Computing Fusionopolis, 1 Fusionopolis Way, 16-16 Connexis, 138632, Singapore; 3 Department of Genetics, University of Santiago de Compostela, ES-27002 Lugo, Galiza, Spain; 4 MRC Human Genetics Unit, Western General Hospital, Crewe Road, Edinburgh, EH4 2XU, UK

## Abstract

**Background:**

It has been shown that if genetic relationships among individuals are not taken into account for genome wide association studies, this may lead to false positives. To address this problem, we used Genome-wide Rapid Association using Mixed Model and Regression and principal component stratification analyses. To account for linkage disequilibrium among the significant markers, principal components loadings obtained from top markers can be included as covariates. Estimation of Bayesian networks may also be useful to investigate linkage disequilibrium among SNPs and their relation with environmental variables.

For the quantitative trait we first estimated residuals while taking polygenic effects into account. We then used a single SNP approach to detect the most significant SNPs based on the residuals and applied principal component regression to take linkage disequilibrium among these SNPs into account. For the categorical trait we used principal component stratification methodology to account for background effects. For correction of linkage disequilibrium we used principal component logit regression. Bayesian networks were estimated to investigate relationship among SNPs.

**Results:**

Using the Genome-wide Rapid Association using Mixed Model and Regression and principal component stratification approach we detected around 100 significant SNPs for the quantitative trait (p<0.05 with 1000 permutations) and 109 significant (p<0.0006 with local FDR correction) SNPs for the categorical trait. With additional principal component regression we reduced the list to 16 and 50 SNPs for the quantitative and categorical trait, respectively.

**Conclusions:**

GRAMMAR could efficiently incorporate the information regarding random genetic effects. Principal component stratification should be cautiously used with stringent multiple hypothesis testing correction to correct for ancestral stratification and association analyses for binary traits when there are systematic genetic effects such as half sib family structures. Bayesian networks are useful to investigate relationships among SNPs and environmental variables.

## Background

It has been shown that ignoring genetic relationships among cases and controls may lead to false positives in genome wide association analyses (GWAS). Genome-wide Rapid Association using Mixed Model and Regression, GRAMMAR, (Aulchenko et al, 2007) approach is a one solution based on correction of quantitative trait for both polygenic and fixed effects. Another approach, introduced by Price et al (2006), uses principal components loadings (PCL) as covariate in linear regression models for detecting and correcting ancestral stratifications for categorical traits in GWAS.

GWAS analyses result in a list of significant SNPs. Some of these SNPs will be in linkage disequilibrium (LD) due to the dense set of markers and this may lead to collinearity among these SNPs. Collinearity among genotype scores could raise problems when applying model selection procedures and different solutions have been proposed from non parametric methods to selective algorithms (Wang and Abbott, 2008). Wang and Abbott (2008) suggested using a principal component regression (PCReg) approach to break the collinearity among marker genotypes using top principal components loadings (PCL) as covariates in the linear regression model. Pant et al (2010) extended the PCReg approach to the categorical traits via logistic regression and model selection strategies.

Most of the GWAS studies do not take into consideration the possible relations among SNPs and/or with other explanatory variables. Bayesian networks are models that present statistical dependencies and independencies in the joint probability distribution of the data. As such they do not state causality, but it is certainly possible to speculate what kind of causal mechanisms would be compatible with the observed dependencies and independencies. Rodin et al (2005) used Bayesian networks to do joint inference on marker variation in the human APOE gene and plasma apolipoprotein E levels. Sebastiani et al (2005) used Bayesian networks in association studies.

The main aim of this study was to apply GRAMMAR, Bayesian Network and principal component stratification models to QTL-MAS 2010 dataset.

## Methods

### Genome-wide rapid association using mixed model and regression

In the first step of the GRAMMAR analysis, we estimated the heritabilities and residuals for the quantitative trait using an animal model as was implemented in Asreml (http://www.vsni.co.uk/software/asreml);

y = **X**b + **Z**a + e (1)

, where y contains the observations, b is the fixed effects of sex, a is the additive genetic effect, matrices **X** and **Z** are incidence matrices, and **e** is a vector containing residuals.

For the random effects, it is assumed that **A** is the additive genetic relationship matrix for the animals; **I** is an identity matrix**,** is the additive genetic variance and  is the residual variance. In the second step, assuming a single SNP model for the quantitative trait, we could detect the most significant SNPs using the following model:

**y**=**X**f + **η** + **e** (2)

where **y** represents vector of *n* observations (residuals from (1)), **η** is intercept, **X** is a design matrix relating observations with **f** regression coefficients vector to be estimated, **e** is a vector of residuals assumed to be normally distributed. We applied GRAMMAR (Aulchenko et al, 2007) with 1000 permutation to detect most significant SNPs for quantitative trait using residuals obtained (1) and used in (2) as response variable.

### Principal components analyses

Principal components analyses can be used to decompose the genomic matrix into a set of new orthogonal variables which account for the total variance of the original variables (Everitt et al., 2001) in decreasing proportions. For the binary trait, we used principal component stratification (Price et al, 2006) methodology to account for ancestral stratification in the QTL-MAS2010 dataset, as was implemented in SAS and JMP Genomics software (SAS institute, Inc, Carey, NC, USA) version 9.1. In order to take LD among significant markers into account for GRAMMAR and principal component stratification approaches we applied principal component analyses with 21 (PCL) as covariate (about 80% of variance explained) for the quantitative trait and 20 PCL as covariate (about 75% of variance explained) for the binary trait in regression models (Minitab, Ver 14).

### Bayesian network

Bayesian networks are multivariate models for determining the probability of an n-dimensional discrete data vector X=(X_1_, …, X_n_) (Pearl, 1988). Bayesian networks consist of two components: a directed acyclic graph G=(G_1_, …, G_n_) and the parameters Θ=(Θ_1_, …, Θ_n_). The graph G determines for each variable X_i_ a set G_i_ of parent variables (i.e. variables from which there are directed arcs to X_i_). A hypothesis conveyed by such a graph is that the probability of vector X can be expressed as a product of conditional probabilities parameterised by the components of Θ:

For data D of n-dimensional i.i.d. data vectors the formula above allows us to calculate the probability P(D | G, Θ). More interestingly, in a Bayesian setting, under certain technical assumptions, after giving a prior distribution for the parameters Θ, one may calculate the marginal likelihood P(D | G,α), where α denotes the hyperparameters for the prior distribution of the Θ. This allows us to compare Bayesian network structures by their posterior probability P(G | D,α) α P(D | G,α)P(G) (Heckermann et al. 1995). It is well known that finding the most probable Bayesian network structure is an NP-hard problem (Chikering 2002). Therefore it is customary to resort to search heuristics such as a local greedy search. However, it has been shown that the most probable forest structured Bayesian network (i.e. a network in which each variable has at most one parent) can be found in quadratic time with respect to number of variables n (Heckermann et al. 1995).

We used the significant markers found from GRAMMAR and principal component approaches to train the Bayesian network. We learned both general Bayesian network and Bayesian Forests using different search algorithms to compare the results with LD measures. Details of the LD measures used in this paper could be found in Devlin and Risch (1995).

## Results

### Quality Control

We excluded 263 SNPs due to minor allele frequency <1%, leaving 9768 SNPs in the analyses. We excluded 8 individuals with too high IBS (Identity By State) (>95%) leaving 2318 individuals in the dataset. Normality for the quantitative trait was confirmed by Kolmogorow Smirnow test, P > 0.150. Based on an animal model (1) we estimated heritabilities as 0.44 (±0.05) for the binary trait and 0.58 (±0.12) for the quantitative trait.

### Analysis of binary trait

We used 20 PCL to take possible ancestral stratification into account for the binary trait. Visual inspection of the scree plot showed that the sharpest reduction was obtained from the first 10 principal components. We detected the top 109 SNPs based on local FDR (Strimmer, 2008a) (p<0.0006). In order to take linkage disequilibrium into account we additionally applied PCReg with normal and logit functions. Using the first 20 PCL as covariate, we applied PCReg to 109 markers with a logit function, and obtained the top 50 SNPs from this approach. We also investigated loading plots for the top SNPs. Although some clusters were found related with location of the SNPs and LD among them, this was not observed consistently for the entire genome (Additional File 1). We mapped 5 QTL’s correctly with 0.33Mb average distance from the simulated QTL. Although top markers such as 5488 or L4483 were detected by the model, 41 QTL showed an average distance of 5.5 Mb from the nearest real QTL and could be considered false positives. This suggests that under the strong systematic genetic effects (as such as a half-sib family structure) more stringent multiple hypothesis testing correction procedures should be used.

### Analysis of quantitative trait

**Figure 1:**
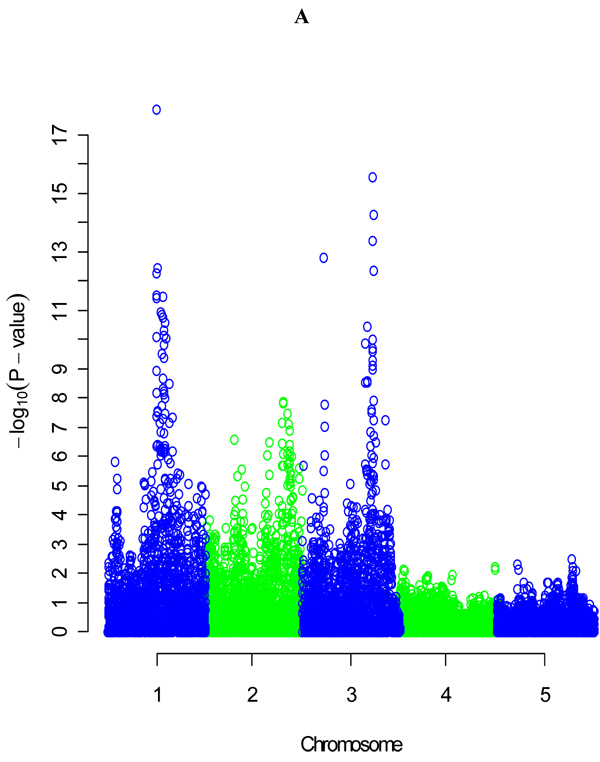
GRAMMAR results for quantitative trait without (A).

**Figure 2:**
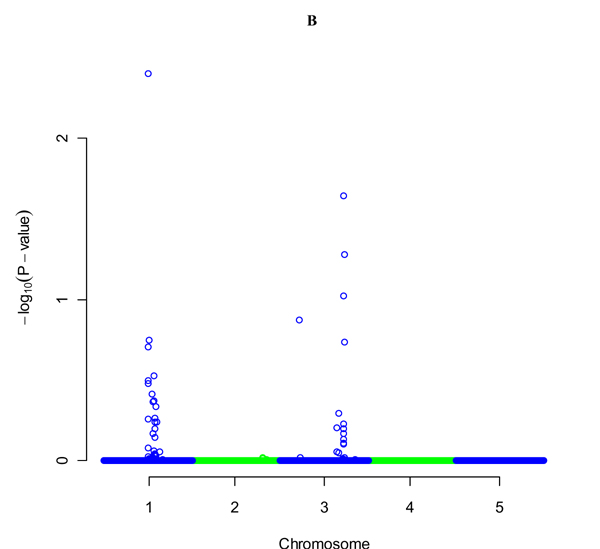
GRAMMAR results for quantitative trait with 1000 permutations (B).

Using Grammar we obtained the top 106 SNPs with 1000 permutation (p<0.05) (Figure 1 and 2). We used PCReg with 21 PCL to take collinearity among them into account and reduced the list to 16 SNPs. We also investigated loading plots for top the SNPs. Again some clusters showed a relation with locations of SNPs and LD among them, but this was not observed consistently for the entire genome. We mapped 5 QTLs with a mean distances of 0.31 Mb from the simulated QTL mean distances. We found 7 false positives with 3.6 Mb mean distances.

### Bayesian network

We constructed the general Bayesian Networks using non-informative priors and did not use any prior biological information (Myllymaki et al, 2002)(Additional file 2). We used significant markers from GWAS to estimate the Bayesian Networks. The learned network was compared empirically with the LD statistics and arc strengths. This showed incomplete concordance between the two approaches (Table 1). Because the Bayesian network is a multivariate model and LD measures are pair-wise only differences can arise (Table 1). Subsequently, we created the Bayesian Forest (Appendix 2), which is also pair-wise, and compared some of the nodes with LD measures (Table 2). Arc strengths showed relative similarities with correlation, Yules Q and D prime LD measures (Table 2). We estimated Pearson correlations among the common SNPs from both methodologies, which were higher for Bayesian Forest (Table 3 and Additional File 3).

**Table 1:** Comparison of linkage disequilibrium measures with general Bayesian Network arc strengths.

Marker1	Marker2	Chi	P(Chi)	D	CorrCoeff	Dprime	Delta	PropDiff	YulesQ	ARC	exp(ARC)
A8111	A9100	692.12	1.50x10^-152^	0.09	0.55	0.87	0.88	0.71	0.95	293.53	3.01x10^127^
A8363	A9100	516.77	2.10x10^-114^	0.08	0.47	0.99	0.99	0.66	0.99	71.68	1.35x10^31^
A8111	A8363	548.72	2.40 x10^-114^	0.11	0.49	0.64	0.51	0.45	0.82	519.43	3.85x10^225^
A8111	A8351	2318	1.70 x10^-236^	0.16	0.68	0.98	0.63	0.63	0.99	694.50	4.14x10^301^
A8035	A8329	1668.98	0	0.21	0.85	0.97	0.84	0.83	1.00	232.66	1.10x10^101^
A8329	A8351	20.12	7.27 x10^-6^	-0.02	-0.09	-0.11	-0.19	-0.09	-0.19	240.85	3.98x10^104^

Arc (and it is exponent) shows that taking the arc away from the current network would make the resulting model less probable; hence bigger arc number shows stronger association.

D Linkage Disequilibrium Coefficient

CorrCoeff: Correlation coefficient

Dprime: Lewontin’s *D’*

Delta: Population attributable risk, δ

PropDiff: Proportional difference

YulesQ: Yule’s Q

**Table 2:** Comparison of Bayesian Forest estimates with Linkage Disequilibrium estimates.

Marker1	Marker2	ChiSq	ProbChi	D	CorrCoeff	Dprime	Delta	PropDiff	YulesQ	ARC	Exp(ARC)
A599	A613	1567.399	0	0.09	0.82	0.95	0.74	0.74	1.00	850.271	NA*
A599	A5603	117.2745	2.50 x10^-27^	0.04	0.22	0.60	0.15	0.14	0.66	80.5	9.13 x10^34^
A3102	A3105	1916.852	0	0.20	0.91	1.00	1.00	0.94	1.00	1668.336	NA*
A3102	A3444	128.9518	6.95 x10^-30^	0.03	0.24	0.66	0.67	0.46	0.76	80.14	6.37 x 10^34^

*Number is too big to show in the table.

**Table 3:** Pearson correlations between General Bayesian network (A) and Bayesian Forest (B) and linkage disequilibrium measures.

***A***	D	Correlation Coefficients	D Prime	Yules Q
**Correlation Coefficients**	**0.961**			

**D prime**	**0.689**	**0.776**		

**Yules Q**	**0.719**	**0.809**	**0.950**	

**ARC**	**0.692**	**0.726**	**0.483**	**0.456**

***B***	**D**	**Correlation Coefficients**	**D Prime**	**Yules Q**

**Correlation Coefficients**	**0.882**			

**D prime**	**0.434**	**0.601**		

**Yules Q**	**0.537**	**0.697**	**0.900**	

**ARC**	**0.892**	**0.915**	**0.576**	**0.569**

### Estimation of SNPs effects

For quantitative trait we estimated the SNPs effects with linear models. We used residuals from model (1) as response variable in a linear model. When we used phenotypes as response variable linear model tend to overestimate the total explanatory variation. We estimated QTL variance for the top marker (5488) as 7.7 % and 2.9 % using linear models with phenotypic and residual values, respectively. This QTL was simulated with 4.49 % variance suggesting that using residuals gave more correct estimates. When residuals are not normally distributed, orthogonal models are robust compared with a linear model from deviation of normality (Sarabia et al, 1997).

## Conclusions

When cases and controls have genetic relations, GRAMMAR could efficiently incorporate the information regarding random genetic effects. Principal component stratification could be used to correct for ancestral stratification and association analyses for binary traits although if there is systematic genetic effects stringent multiple hypothesis test corrections should be used. Bayesian networks are useful to investigate relationships among SNPs and environmental variables. Although a learned network does not have to show causal relationships, it is still informative and creates hypotheses based on interactions among SNP’s.

## Competing interests

The authors declare that they have no competing interests.

## Authors' contributions

BK compiled the dataset and done genetic, GRAMMAR, principal component stratification and PCReg analyses. TS and BK estimated the Bayesian networks. BK and TS wrote the manuscript. JAC, CH and DJK advised on analysis and data interpretation and revised the manuscript.

## Supplementary Material

Additional file 1 **Loadings of first 2 principal component of binary trait from top 109(AXX) markers using principal component stratification model. Although some markers cluster according to high linkage disequilibrium and by chromosome, this is not consistently true over the genome.** Loadings of first 2 principal component of binary trait from top 109(AXX) markers using principal component stratification model. Although some markers cluster according to high linkage disequilibrium and by chromosome, this is not consistently true over the genome. Click here for file

Additional files 2 **Learned general Bayesian network for binary trait using top 109 markers obtained from principal component stratification methodology.** Learned general Bayesian network for binary trait using top 109 markers obtained from principal component stratification methodology. Click here for file

Additional file 3 **Learned Bayesian Forest for binary trait using top 109 markers obtained from principal component stratification methodology.** Learned Bayesian Forest for binary trait using top 109 markers obtained from principal component stratification methodology. Click here for file
