# Supplementary material for: Association analyses of the MAS-QTL data set using grammar, principal components and Bayesian network methodologies
Source: BMC Proc. 2011 May 27;5(Suppl 3):S8. doi: 10.1186/1753-6561-5-S3-S8 (PMC3103207; doi:10.1186/1753-6561-5-S3-S8)
Supplement: Additional file 1 — Loadings of first 2 principal component of binary trait from top 109(AXX) markers using principal component stratification model. Although some markers cluster according to high linkage disequilibrium and by chromosome, this is not consistently true over the genome. Loadings of first 2 principal component of binary trait from top 109(AXX) markers using principal component stratification model. Although some markers cluster according to high linkage disequilibrium and by chromosome, this is not consistently true over the genome. [file 1753-6561-5-S3-S8-S1.doc]

**Additional files_1**

**1 File Name**

Additional Figure 1 Loadings of first 2 principal component of binary trait from top 109(AXX) markers using principal component stratification model. Although some markers cluster according to high linkage disequilibrium and by chromosome, this is not consistently true over the genome.
