# Supplementary material for: Association analyses of the MAS-QTL data set using grammar, principal components and Bayesian network methodologies
Source: BMC Proc. 2011 May 27;5(Suppl 3):S8. doi: 10.1186/1753-6561-5-S3-S8 (PMC3103207; doi:10.1186/1753-6561-5-S3-S8)
Supplement: Additional file 3 — Learned Bayesian Forest for binary trait using top 109 markers obtained from principal component stratification methodology. Learned Bayesian Forest for binary trait using top 109 markers obtained from principal component stratification methodology. [file 1753-6561-5-S3-S8-S3.doc]

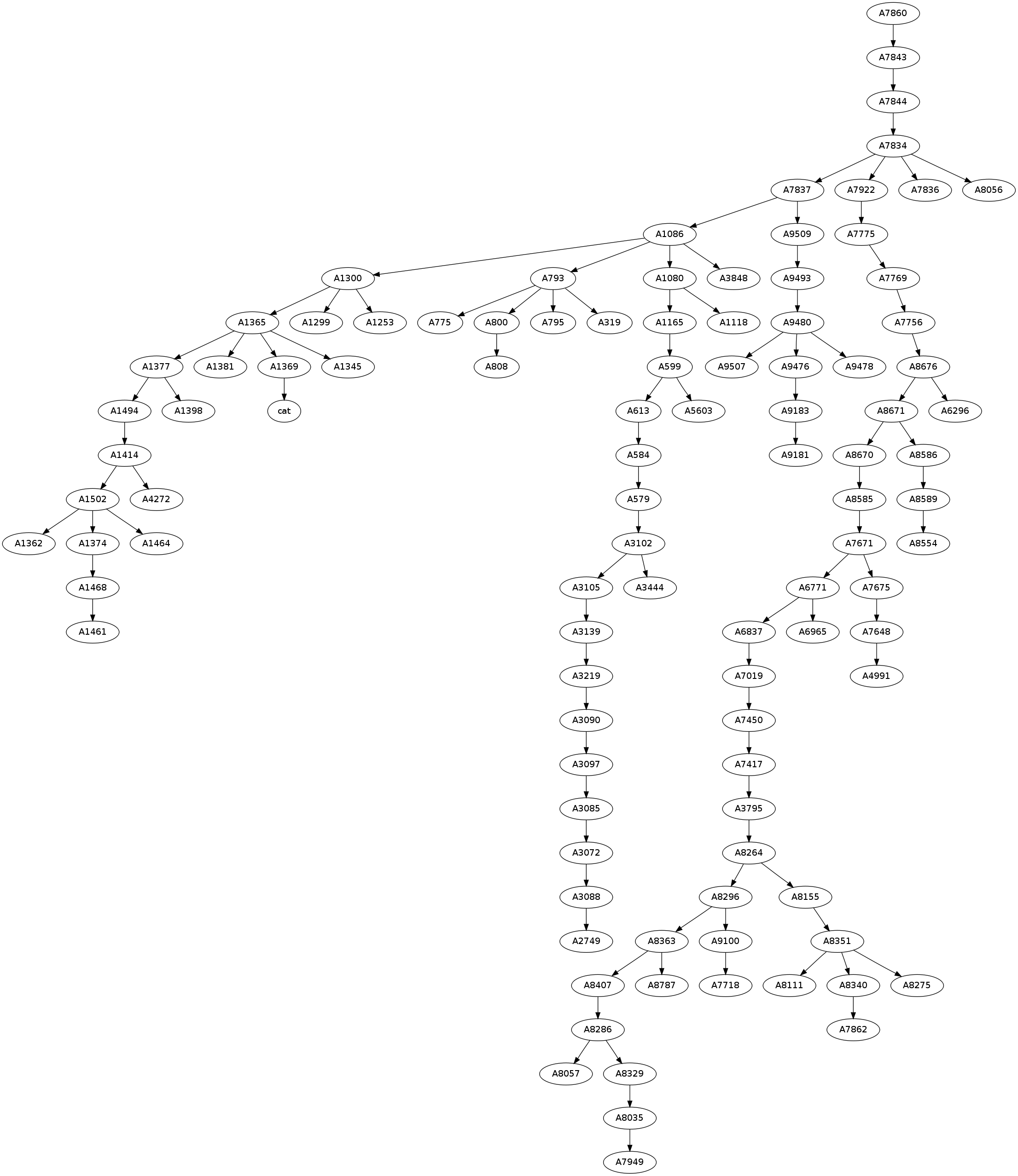


Additional file 3 – Learned Bayesian Forest for binary trait using top 109 markers obtained from principal component stratification methodology.
